# CircPrimer 2.0: a software for annotating circRNAs and predicting translation potential of circRNAs

**DOI:** 10.1186/s12859-022-04705-y

**Published:** 2022-06-06

**Authors:** Shanliang Zhong, Jifeng Feng

**Affiliations:** 1 Center of Clinical Laboratory Science, The Affiliated Cancer Hospital of Nanjing Medical University & Jiangsu Cancer Hospital & Jiangsu Institute of Cancer Research, Nanjing, 210009 China; 2 Department of Medical Oncology, The Affiliated Cancer Hospital of Nanjing Medical University & Jiangsu Cancer Hospital & Jiangsu Institute of Cancer Research, Baiziting 42, Nanjing, 210009 China

## Abstract

**Background:**

Some circular RNAs (circRNAs) can be translated into functional peptides by small open reading frames (ORFs) in a cap-independent manner. Internal ribosomal entry site (IRES) and N^6^-methyladenosine (m^6^A) were reported to drive translation of circRNAs. Experimental methods confirming the presence of IRES and m^6^A site are time consuming and labor intensive. Lacking computational tools to predict ORFs, IRESs and m^6^A sites for circRNAs makes it harder.

**Results:**

In this report, we present circPrimer 2.0, a Java based software for annotating circRNAs and predicting ORFs, IRESs, and m6A sites of circRNAs. circPrimer 2.0 has a graphical and a command-line interface that enables the tool to be embed into an analysis pipeline.

**Conclusions:**

circprimer 2.0 is an easy-to-use software for annotating circRNAs and predicting translation potential of circRNAs, and freely available at www.bio-inf.cn.

**Supplementary Information:**

The online version contains supplementary material available at 10.1186/s12859-022-04705-y.

## Background

Circular RNAs (circRNAs) are a family of regulatory RNAs with loop structures which implies they do not have 5`Caps and 3` Poly (A) tails [1]. Although a great number of circRNAs have been identified, their functions are still largely unknown. CircRNAs are generally considered noncoding RNAs with various biological functions. Up to now, the vast majority of studies that investigated function of circRNAs have been based around the miRNA-sponge activity of these molecules [2]. Nevertheless, some studies reported that circRNAs can be translated into functional peptides by small open reading frames (ORFs) [3]. Since circRNAs do not have 5` Caps, circRNAs cannot be translated in a cap-dependent manner. Two mechanisms have been reported to initiate translation of circRNAs. First, internal ribosomal entry site (IRES) recruits ribosomes to the internal site of circRNA to initiate translation [3]. Second, N^6^-methyladenosine (m^6^A) drives translation with the help of initiation factor eIF4G2 and m^6^A reader YTHDF3 [4, 5]. Therefore, the existence of ORF and IRES or m^6^A site is a prerequisite to encode peptides for a circRNA. However, experimental methods confirming the presence of IRESs and m^6^A modification sites are time consuming and labor intensive [5, 6]. Lacking computational tools to predict IRESs and m^6^A sites as well as ORFs for circRNAs makes it harder. At present, no tool predicts ORFs, IRESs or m^6^A modification sites specificity for circRNAs.

Here, we present circPrimer 2.0, a user-friendly software to help researchers study circRNAs. We rewrote all codes of former version of circPrimer [7]. CircPrimer 2.0 includes all features of former version, with optimized performance. Besides annotating circRNAs and determining specificity of circRNA primers, circPrimer 2.0 can show conserved circRNAs, and predict ORFs, IRESs and m^6^A modification sites. The results are presented visually and can be saved as PDF format. CircPrimer 2.0 also provides command-line interface, therefore it can be integrated into analysis pipelines.

## Implementation

### Prediction of ORFs

To predict ORFs for a circRNA, the start codons and stop codons are searched for each frame. When two or more start codons are found in the upstream of a stop codon in a frame, we choose the one far from the stop codon as the start codon. Studies have reported that circRNA containing an infinite ORF can be efficiently translated to produce a long-repeating peptide sequence [8, 9], thus we also predict infinite ORFs. The accuracy of ORF prediction were evaluated using ORFfinder (Linux × 64; www.ncbi.nlm.nih.gov/orffinder/).

**Fig. 1:**
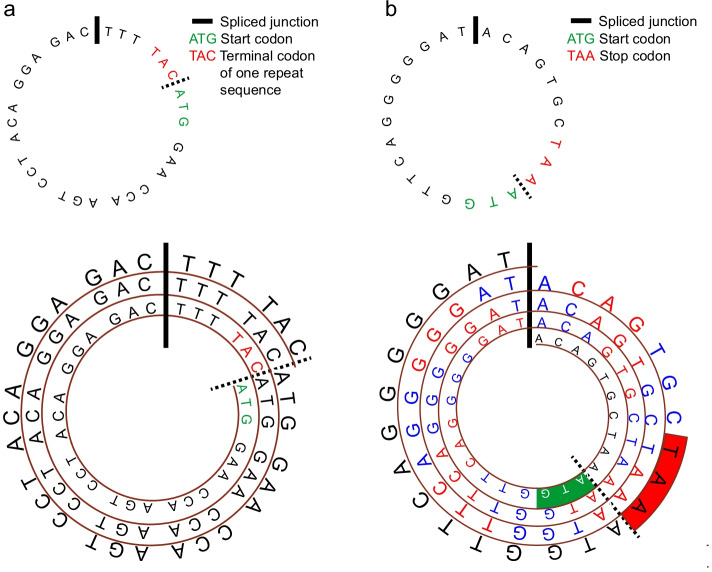
The methods used to predict open reading frames (ORFs) and internal ribosomal entry sites (IRESs). **a** Predicting ORFs for a circRNA with a length that can be evenly divided by three. **b** Predicting ORFs for a circRNA with a length that cannot be evenly divided by three

There are two situations in predicting ORFs for circRNAs. The first one is that the sequence length of a circRNA can be evenly divided by three. Figure 1a presents an example of this type of circRNA. In this situation, the frame will not shift in rolling circle translation. If there is a stop codon in a frame, the maximum length of an ORF is equal to circRNA length. If an infinite ORF is found in a frame, the frame may produce a long-repeating peptide sequence in a manner of rolling circle translation (Fig. 1a). The full sequence of the circRNA from the start codon down to the terminal codon comprises one rolling circle translation.

The second situation is that the circRNA length cannot be evenly divided by three. When an ORF spans the back-spliced junction, the frame will shift. When there is a stop codon in a frame, the maximum length of an ORF in this frame is equal to 3-folds of circRNA length (Fig. 1b). If an infinite ORF is found in a frame, the length of one repeat sequence from the start codon down to the terminal codon is also equal to 3-folds of circRNA length.

### Prediction of IRES

To predict IRES, we used TGBoost package (https://github.com/wepe/tgboost) to build the models for IRES predication with the 20,872 native IRES sequences reported by Gritsenko et al. [10]. Wang et al. have demonstrated that using global kmer features only can obtain high prediction performance [6], thus we established our models using global kmer features. We randomly divided the data into training (90%) and test dataset (10%) and used tenfold cross validation to evaluate each combination of parameters. The best fit parameters were summarized to generate the final set of model parameters.

Wang et al. divided the kmer count by the sequence length to remove the influence of sequence length [6]. However, we found that an IRES in a long sequence will obtain a negative result. That is because the kmer features are diluted by the long none IRES sequence. Therefore, we split the full circRNA sequence into fragments of 174 nt, which is equal to Gritsenko et al.’ data [10]. The step used to split the sequence is 20 nt, i.e. every two consecutive fragments with a 154-base overlap. Then the kmer frequencies are calculated for each fragment. If 2 or more fragments are predicted as IRES, the IRES near the start codon is considered as the IRES of an ORF. It should be noted that a positive result does not mean the 174 nt fragment is IRES but the fragment contains an IRES. Command-line interface can be used to predict IRESs with shorter fragments.

### Dataset of m^6^A modification sites

We downloaded m^6^A modification sites for Human and Mouse from m^6^A-Atlas [11]. m^6^A-Altas is a comprehensive knowledgebase for unraveling the m^6^A epitranscriptome, which features a high-confidence collection of reliable m^6^A sites identified from seven base-resolution technologies and the quantitative condition-specific epitranscriptome profiles estimated from high-throughput sequencing samples. Because the reference genome of the m^6^A sites is hg19 for Human and GRCm38.p6 for Mouse, we transformed hg19 to hg38 and GRCm38.p6 to mm9 using Remap (www.ncbi.nlm.nih.gov/genome/tools/remap) for genomic locations in hg38 and mm9.

Because m^6^A modification occurs within the consensus DRACH/RRACH motifs [5, 12, 13], circPrimer 2.0 shows all DRACH/RRACH motifs (D = A, G or U; R = G or A; H = A, C or U) for a sequence.

### Identification of homeotic circRNA

We identified homeotic circRNAs between Homo sapiens and Mus musculus using the following criteria: (1) The circRNAs are derived from same gene; (2) Their sequence length is identical; and (3) The identity of their sequences is larger than 80%.

## Results

### Features of circPrimer 2.0

CircPrimer 2.0 is written in Java and provides both a graphical and command-line interface. Compared with circPrimer 1.2, circPrimer 2.0 can (1) Predict ORFs and IRESs for all circRNAs with their sequences; (2) Be integrated into analysis pipelines; (3) Show conserved circRNAs and identities between Homo sapiens and Mus musculus; (4) Run in all platforms, including Window, Mac OS X, Linux, and Solaris; (5) Search and annotate circRNAs more quickly; (6) Export data in different formats, Fasta, txt, or csv; (7) Save figures in PDF format; and (8) Search and annotate circRNAs of Mus musculus. Because we used cloud database to store our data, the size of circPrimer is compressed from 3G to 4 M.

### Evaluating ORF prediction accuracy

We randomly selected 1000 sequences from circBase, and predicted ORFs using ORFfinder and circPrimer 2.0. Because ORFfinder is unable to predict ORFs for circRNAs, their results cannot be compared directly. First, we removed the ORFs spanning the back-spliced junctions for circPrimer 2.0. Second, we filtered the ORFs without a stop codon for ORFfinder. Third, we compared the rest ORFs with each other. We found that the rest ORFs of circPrimer 2.0 were identical to those of ORFfinder (Additional file 1: Data S1).

**Fig. 2:**
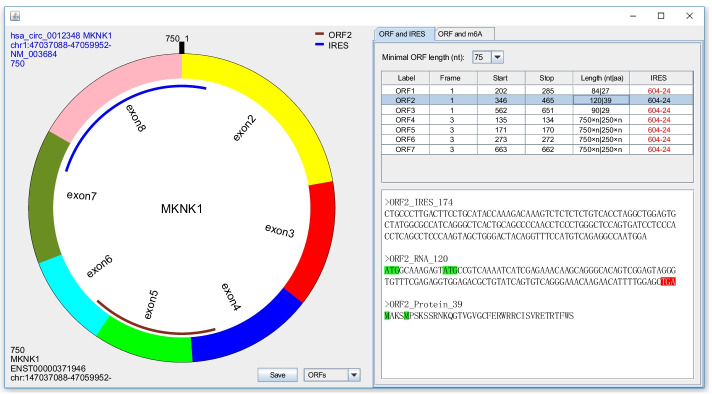
Predicted open reading frames (ORFs) and internal ribosomal entry sites (IRESs) are shown visually. ORF4 ~ ORF6 are infinite ORFs which lack a stop codon and are labeled with “a number × n” in the Length field. The number is the length of one repeat sequence. Green background, start codons in the same frame of the ORF; red background, stop codon. Red font in IRES field, IRESs spanning back-spliced junctions

Because Legnini et al. reported that a start codon, in the same frame, presented in the downstream of the first one can also drive translation [14], circPrimer 2.0 highlights these inner start codons with green background (Fig. 2).

### Building models for IRES prediction and performance evaluation

The tuning parameters of TGBoost model showed that the optimal parameters are eta = 0.03, max_depth = 5, scale_pos_weight = 8.78, subsample = 0.9, colsample_bytree = 0.5, min_child_weight = 19, gamma = 0, lamda = 1, alpha = 0. To test accuracy in circRNAs, we searched PubMed for the studies reported coding circRNAs and obtained 10 human circRNAs [14–23]. Because one study did not reported detailed information, we have failed to obtain their circRNA sequence [16]. Another study did not assess the translation initiation mechanisms [21]. Therefore, the two studies were removed. We used circPrimer 2.0 to predict ORFs for the rest 8 circRNAs. All ORFs were predicted by circPrimer 2.0. When predicting IRESs, it failed to find an IRES site in 3 circRNAs [15, 17, 22], and predicted at least one IRES in the other 5 circRNAs, showing a sensitivity of 63% (Additional file 2: Table S1).

We also assessed the performance of the model using test dataset. The accuracy predicting IRESs is 74.1%, sensitivity is 64.8% and specificity is 75.1%.

### Showing the predicted ORFs, IRESs and m^6^A modification sites

After searching or annotating circRNAs or checking primers, the circRNAs will be listed in the middle panel. When clicking one item, a dialog will show the circRNA structure. If you set ComboBox as “ORFs”, a right panel will show the predicted ORFs and IRESs. “None” in the field of IRES means none IRES is found in this circRNA; otherwise, the positions of IRESs are shown. Because IRESs spanning back-spliced junctions may exhibit a splicing dependent IRES activity [24], circPrimer 2.0 highlights these IRESs with red font (Fig. 2). You can click one item to show an ORF and its IRES visually as well as their detailed information (Fig. 2). If you select two or more items, only ORFs are shown visually. To indicate an infinite ORF, the length of the ORF will be labeled with “a number × n”. The number is the length of one repeat sequence (Fig. 2).

The panel of “ORF and m6A” shows the m^6^A modification sites.

### Showing homeotic gene

After comparing circRNA sequences between Homo sapiens and Mus musculus, we obtained 3439 paired conserved circRNAs. The conserved circRNAs are shown in red font in the middle panel. When clicking one conserved circRNA, the identity of the sequences between Homo sapiens and Mus musculus will show in the right-bottom textarea.

## Discussion

In the present study, we present circPrimer 2.0, a Java based software for annotating circRNAs and predicting ORFs and IRESs of circRNAs. At present, circRNADb and Circbank had predicted ORFs and IRESs for a number of circRNAs [25, 26]. Because Circbank only shows the ORF size and locations of IRESs, users are unable to obtain ORF locations or sequences, and users have to extract IRES sequences manually. In addition, Circbank used IRESfinder [27] to predict IRESs, which has been reported to have some obvious shortcomings [6]. circRNADb predicted IRESs using VIPS, a tool for predicting viral IRESs [28]. Both tools are unable to show ORFs and IRESs for novel circRNAs. Therefore, circPrimer 2.0 is the first tool specifically designed to predict ORFs and IRESs of circRNAs.

## Conclusions

We demonstrated the reliability of circPrimer 2.0 in predicting ORFs and IRESs. CircPrimer 2.0 shows the positions of ORFs, IRESs and m^6^A sites visually. Users can perform the predication with preferred parameters using command-line interface. CircPrimer 2.0 shows conserved circRNAs and identities between Homo sapiens and Mus musculus. In summary, circPrimer 2.0 is an easy-to-use software annotating circRNAs and predicting translation potential of circRNAs.

## Availability and requirements

Project name: circPrimer 2.0

Project home page: www.bio-inf.cn

Operating system(s): Window, Mac OS X, Linux, and Solaris

Programming language: Java

Other requirements: Internet connectivity and Java 1.8.0 or higher

License: GNU General Public License version 3.0 (GPL-3.0)

Any restrictions to use by non-academics: None.

## Supplementary Information

**Additional file 1:** Python and R scripts as well as the data generated to evaluate the accuracy of circPrimer 2.0 in predicting open reading frames.

**Additional file 2:** Testing accuracy of circPrimer 2.0 in predicting IRESs and ORFs in reported coding circRNAs.

## Data Availability

The datasets analysed during the current study are available in the Bitbucket repository, https://bitbucket.org/alexeyg-com/irespredictor.

## Abbreviations

circRNA: Circular RNA
ORF: Open reading frame
IRES: Internal ribosomal entry site
